# Institutional Perspectives on Assisted Reproductive Technology Using Third‐Party Gametes in Japan: A Nationwide Survey of Assisted Reproductive Technology Facilities

**DOI:** 10.1002/rmb2.70108

**Published:** 2026-11-03

**Authors:** Nozomi Takahashi, Miyuki Harada, Hiroshi Koike, Yuma Kawahara, Yasuji Kitabatake, Masayuki Kinutani, Tomoe Koizumi, Maki Suganuma, Norihiro Sugino, Nao Suzuki, Akira Tsujimura, Toshio Hamatani, Naoki Mizunuma, Seiji Wada, Yasushi Hirota, Yutaka Osuga

**Affiliations:** ^1^ Department of Obstetrics and Gynecology, Faculty of Medicine The University of Tokyo Tokyo Japan; ^2^ Department of Pediatrics, Graduate School of Medicine The University of Osaka Osaka Japan; ^3^ Kinutani Women's Clinic Hiroshima Japan; ^4^ International Center for Reproductive Medicine Dokkyo Medical University Saitama Medical Center Saitama Japan; ^5^ Department of Psychological and Sociological Studies, School of Cultural and Social Studies Tokai University Tokyo Japan; ^6^ Department of Obstetrics and Gynecology Yamaguchi University Graduate School of Medicine Yamaguchi Japan; ^7^ Department of Obstetrics and Gynecology St. Marianna University School of Medicine Kawasaki City Japan; ^8^ Department of Urology Juntendo University Urayasu Hospital Urayasu Japan; ^9^ Haneda Clinic, Reproduction Center Fujita Health University Tokyo Japan; ^10^ Department of Legal Medicine Toho University Graduate School of Medicine Tokyo Japan; ^11^ Center for Maternal‐Fetal, Neonatal and Reproductive Medicine National Center for Child Health and Development Tokyo Japan; ^12^ Teikyo Academic Research Center Teikyo University Tokyo Japan

**Keywords:** gamete donation, oocyte, sperm, third‐party assisted reproductive technology

## Abstract

**Objective:**

To clarify institutional perspectives on assisted reproductive technology (ART) using third‐party gametes.

**Methods:**

We surveyed 634 facilities registered with the Japan Society of Obstetrics and Gynecology, and 416 responses were included in a descriptive analysis.

**Results:**

Psychosocial and economic stability of ART recipient couples were considered necessary in most facilities (94.6%). Japanese nationality and legal marriage were necessary recipient criteria in 36.8% and 54.3% facilities, respectively. Lower and upper age limits for female recipients were 20–25 and 43–45 years, respectively, whereas most male partners had no upper age limit (34.6%–36.6%). The most common upper age limit for oocyte donors was 35 years (67.9%). In contrast, upper age limits for sperm donors were more dispersed, with 40 years being the most common age at response to intrauterine insemination (35.6%) and in vitro fertilization (35.9%). The most accepted indications were hypergonadotropic hypogonadism for oocyte donation (87.5%) and failure to retrieve sperm via micro‐testicular sperm extraction for donor sperm treatment (97.3%). Most facilities considered psychosocial support necessary.

**Conclusions:**

This study provides a comprehensive overview of institutional perspectives on third‐party ART in Japan. Although facilities showed substantial agreement on core principles, their views on specific eligibility and operational criteria varied.

## 1 Introduction

The application of assisted reproductive technology (ART) has expanded rapidly worldwide, contributing substantially to global birth rates. In Europe and the United States, ART using donated gametes is widely practiced and supported by legal frameworks, registries, and clinical guidance [1, 2]. In Japan, ART use has continued to increase, with 561,664 registered cycles and 85048 neonates reported in 2023, reflecting the recent expansion in public insurance coverage for ART [3]. Despite these advances, a subset of patients remain unable to achieve pregnancy using their own gametes, highlighting the clinical importance of third‐party reproduction, including the use of donor oocytes, sperm, and embryos.

For many years, Japan has lacked a comprehensive legal framework governing ART involving donated gametes. The Act on Special Provisions of the Civil Code Concerning Parent–Child Relationships of Children Born Through Assisted Reproductive Technology came into effect on December 11, 2021; however, the clinical implementation and governance of ART involving third‐party gametes remain incompletely regulated. The Japan Society of Obstetrics and Gynecology (JSOG) highlights that intrauterine insemination (IUI) using donor sperm is acceptable, whereas third‐party embryo donation is prohibited. Consequently, patients seeking third‐party reproductive options may travel abroad to countries such as Taiwan and the United States or pursue treatment through domestic organizations operating under private guidelines. This fragmented landscape underscores the lack of a unified national framework.

Internationally, the regulation and practice of third‐party ART vary considerably depending on legal systems, cultural norms, and religious contexts, with differences in eligibility criteria, donor screening, donor anonymity, and limits of donor use [1, 2, 4]. In Japan, public attitude toward third‐party reproduction and disclosure of donor‐conceived offspring is diverse, and a substantial proportion of the public remains undecided [5]. Additionally, Japanese obstetricians manage pregnancies after oocyte donation, including pregnancies conceived overseas, indicating that the clinical consequences of third‐party reproduction are present in domestic health care [6].

In response to evolving social needs, a bill concerning specified assisted reproductive technologies, including those involving third‐party gametes, was submitted to the Japanese House of Councilors on February 5, 2025; however, this issue has remained unresolved and has not been enacted during any parliamentary session [7]. Given the likelihood of future legislative reconsideration, there is an urgent need to establish evidence‐based, context‐specific guidelines to ensure appropriate implementation of ART using donated gametes in Japan. Although several surveys have assessed public attitudes toward third‐party reproduction in Japan, nationwide investigations targeting ART clinics are limited. This study aimed to assess the attitudes and perceptions of ART facilities nationwide regarding third‐party gamete use.

## 2 Materials and Methods

We conducted a paper‐based survey to assess attitudes toward IUI and in vitro fertilization‐embryo transfer (IVF‐ET) using donor gametes. Among facilities registered with the JSOG, 634 were included in this study; this was based on a search for ART facilities listed on the official JSOG website as of September 2025. These facilities were selected as a target population for questionnaire distribution.

The questionnaire comprised the following domains: (1) basic institutional characteristics; (2) current implementation status of specified ART using third‐party gametes; (3) institutional perspectives regarding recipients of third‐party gametes; (4) institutional perspectives regarding donors; (5) opinions on the operational management of third‐party gametes; and (6) attitudes toward counseling and disclosure (“telling”) practices. The questionnaire was developed as part of a research project funded by the Children and Families Agency, Japan (25DB0201). Its content was developed and refined through multidisciplinary discussions among representatives and experts from relevant professional societies and organizations, including the Japanese Institution for Standardizing Assisted Reproductive Technology (JISART), the Japan Society for Reproductive Medicine, JSOG, the Japanese Urological Association, the Japan Pediatric Society, specialists in reproductive psychology, the National Center for Child Health and Development, legal experts, and other relevant stakeholders. The final questionnaire was determined based on consensus within the research group. The original questionnaire is shown in the Supporting Information. Questionnaires were distributed by mail to the director or designated representative of each eligible ART facility. Accordingly, the responses were considered to reflect the institutional perspectives of the participating facilities. Participation was considered voluntary, and informed consent was obtained using an opt‐in approach, and respondents indicated their agreement by checking a consent box provided in the questionnaire. Only responses accompanied by confirmed consent were included in the analysis. This study was approved by the Ethics Committee of the University of Tokyo Hospital (Approval No. 2025256NI).

The primary analysis was descriptive in nature. Categorical variables were presented as numbers and percentages, and continuous or ordinal variables are presented as medians and ranges, as appropriate. The percentages were calculated using the number of valid responses for each item as the denominator. Exploratory comparisons were performed across four facility types (Table S1) and between facilities with and without reported experience of third‐party reproduction (Table S2). Facilities reporting experience with at least one of the three procedures—IVF using donated oocytes, IUI using donated sperm, or IVF using donated sperm—were classified as having reported experience (*n* = 22). All remaining facilities, including those with unanswered experience items and no affirmative response, were classified as having no reported experience (*n* = 394). Missing responses to individual outcome items were excluded from the corresponding analyses. Continuous variables were compared using the Kruskal–Wallis test across facility types and the Mann–Whitney *U* test between experience groups. Categorical variables were compared using Fisher's exact test for 2 × 2 contingency tables and the Fisher–Freeman–Halton test with Monte Carlo estimation based on 999,999 resamples for larger tables. All applicable tests were two‐sided. To account for multiple comparisons, the Benjamini–Hochberg procedure was applied separately to the 101 tests in each supplementary table. Each multiple‐response option was treated as a separate test, whereas each single‐choice categorical item was evaluated using an omnibus test. Both unadjusted and adjusted *p*‐values are reported; adjusted *p*‐values < 0.05 were considered statistically significant. All comparisons were exploratory and were not adjusted for potential confounders. Statistical analyses were performed using Python with SciPy version 1.18.1.

## 3 Results

### 3.1 Characteristics of Responding Facilities

Among the 634 ART facilities registered with the JSOG and invited to participate, 416 provided consented responses and were included in the analysis (response rate: 65.6%). More than half of the responding facilities were clinics without beds (214/416; 51.4%), followed by clinics with beds (75/416; 18.0%), university hospitals (68/416; 16.3%), and general hospitals (59/416; 14.2%). Annual ART volume varied across facilities: 46 facilities (11.1%) reported < 100 cycles, 129 (31.0%) reported 100–499 cycles, 87 (20.9%) reported 500–999 cycles, and 97 (23.3%) reported ≥ 1000 cycles, 57 (13.7%) facilities did not report annual cycle numbers. Most facilities had 2–4 obstetrician‐gynecologists involved in reproductive medicine (202/416, 48.6%), one reproductive endocrinology and infertility (REI)‐certified physician (178/416, 42.8%), and 2–4 embryologists (228/416, 54.8%) (Table 1). Differences in facility types are listed in Table S1.

**TABLE 1:** Characteristics of participating ART facilities.

	*n* = 416	%
Type of facility		
University hospital	68	16.3
General hospital	59	14.2
Clinic with beds	75	18.0
Clinic without beds	214	51.4
The number of ART cycles		
< 100	46	11.1
100–499	129	31.0
500–999	87	20.9
≥ 1000	97	23.3
No response	57	13.7
The number of Ob/Gyn doctors		
0	0	0.0
1	98	23.6
2–4	202	48.6
5–9	90	21.6
≥ 10	23	5.5
No response	3	0.7
The number of REI‐certified doctors		
0	72	17.3
1	178	42.8
2–4	129	31.0
5–9	30	7.2
≥ 10	2	0.5
No response	5	1.2
The number of embryologists		
0	9	2.2
1	38	9.1
2–4	228	54.8
5–9	87	20.9
≥ 10	45	10.8
No response	9	2.2

### 3.2 Counseling Systems at Responding Facilities

Counseling was available in 341 of 413 facilities (82.6%) (Figure 1A). Counseling availability differed across facility types in the unadjusted analysis, but this difference was not statistically significant after multiple‐comparison adjustment (unadjusted *p* = 0.010; adjusted *p* = 0.108; Table S1). Counseling frequently focused on the provision of medical information (313/341, 91.8%), followed by psychosocial support (247/341, 72.4%) and coordination with healthcare or welfare services (126/341, 37.0%) (Figure 1B). The most common counseling providers were nurses (209/341, 61.3%), physicians (208/341, 61.0%), REI specialists (194/341, 56.9%), and infertility counselors (147/341, 43.1%); certified fertility nurses, clinical psychologists, reproductive counselors, licensed psychologists, and social workers were less frequently involved (Figure 1C).

**FIGURE 1:**
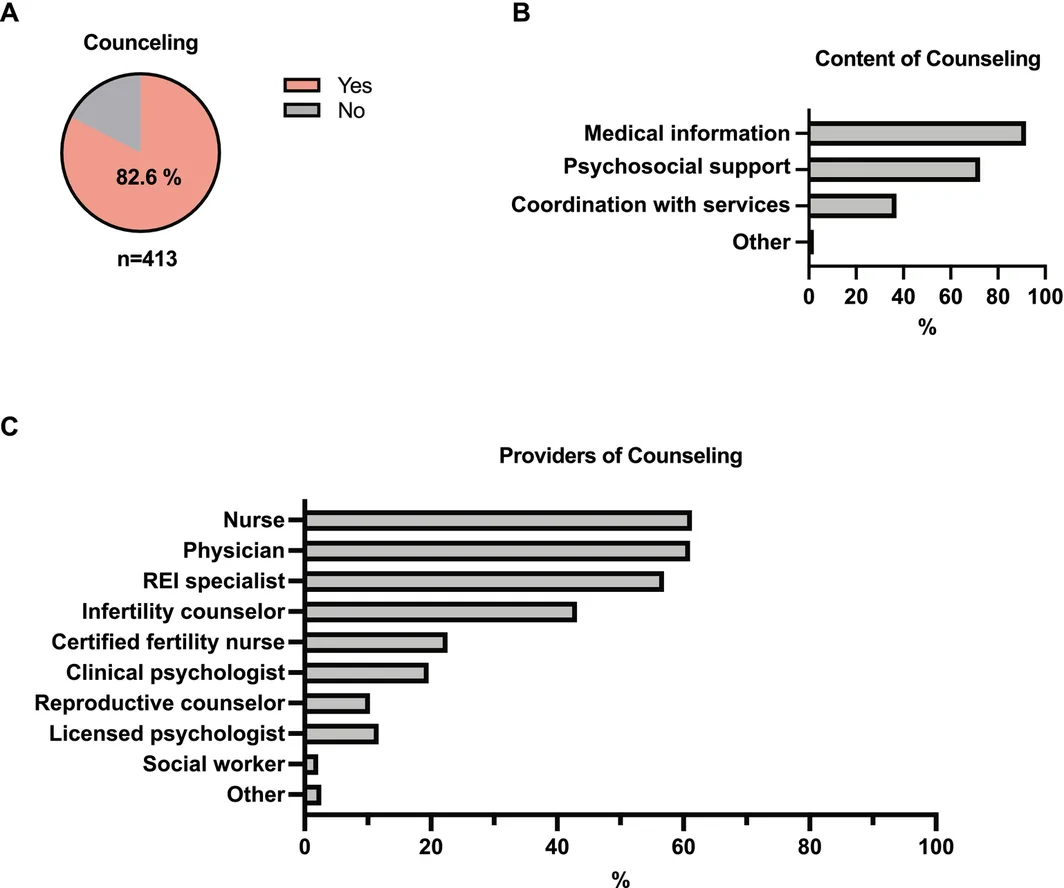
Counseling systems in responding ART facilities. (A) Proportion of facilities providing counseling. (B) Main contents of counseling, including medical information, coordination with health‐care or welfare services, psychosocial support, and other counseling topics. (C) Professional categories involved in counseling. ART, assisted reproductive technology; REI, reproductive endocrinology and infertility.

### 3.3 Current Implementation of Specified ART Using Third‐Party Gametes

Twenty‐two (5.3%) facilities experienced third‐party ART. Oocyte donation for IVF was reported by nine facilities, donor sperm IUI by 14 facilities, and donor sperm IVF by nine facilities. Median number of treatment cases was 10 (range: 1–197) for oocyte donation IVF, 408 (range: 25–17,074) for donor sperm IUI, and 11 (range: 2–271) for donor sperm IVF. Donor age was 22–47 years for oocyte donation, 19–50 years for sperm donation for IUI, and 20–79 years for sperm donation for IVF. Female recipient ages ranged between 22 and 50 years for oocyte donation IVF, 21–50 years for donor sperm IUI, and 22–47 years for donor sperm IVF, whereas male partner ages ranged from 23 to 61, 21 to 64, and 23 to 52 years, respectively (Table 2). Facilities with reported experience had higher annual ART volumes (median, 1403 vs. 492 cycles) and more embryologists (median, 7 vs. 3) than facilities without reported experience (both adjusted *p* = 0.014). No questionnaire items concerning institutional perspectives showed statistically significant differences after adjustment (Table S2).

**TABLE 2:** Third‐party gamete ART.

	Oocyte donation (IVF)	Sperm donation (IUI)	Sperm donation (IVF)
Number of facilities	9	14	9
Number of cases, median (range)	10 (1–197)	408 (25–17,074)	11 (2–271)
Donor age, years (range)	22–47	19–50	20–79
Marital status of donor			
Married	3	0	1
Unmarried	0	4	1
Both experienced	4	7	5
Unknown	2	3	2
Spouse age of donor, years (range)	23–55	25–42	25–71
Female recipient age, years (range)	22–50	21–50	22–47
Male partner age, years (range)	23–61	21–64	23–52

Abbreviations: IUI, intrauterine insemination; IVF, in vitro fertilization.

### 3.4 Views on Recipient Eligibility

The majority of facilities considered psychosocial and economic stability of the ART recipient couple to be necessary (386/408, 94.6%) (Figure 2A). In the questionnaire, “economic stability” was intended to refer to sufficient and stable financial resources to support child‐rearing, without specifying an income threshold. In contrast, only 149 of 405 facilities (36.8%) supported limiting recipients to those of Japanese nationality, whereas 247 (61.0%) did not support such a limitation (Figure 2B). Legal marriage was considered necessary by 221 of 407 facilities (54.3%), whereas 178 (43.7%) did not consider marriage necessary (Figure 2C).

**FIGURE 2:**
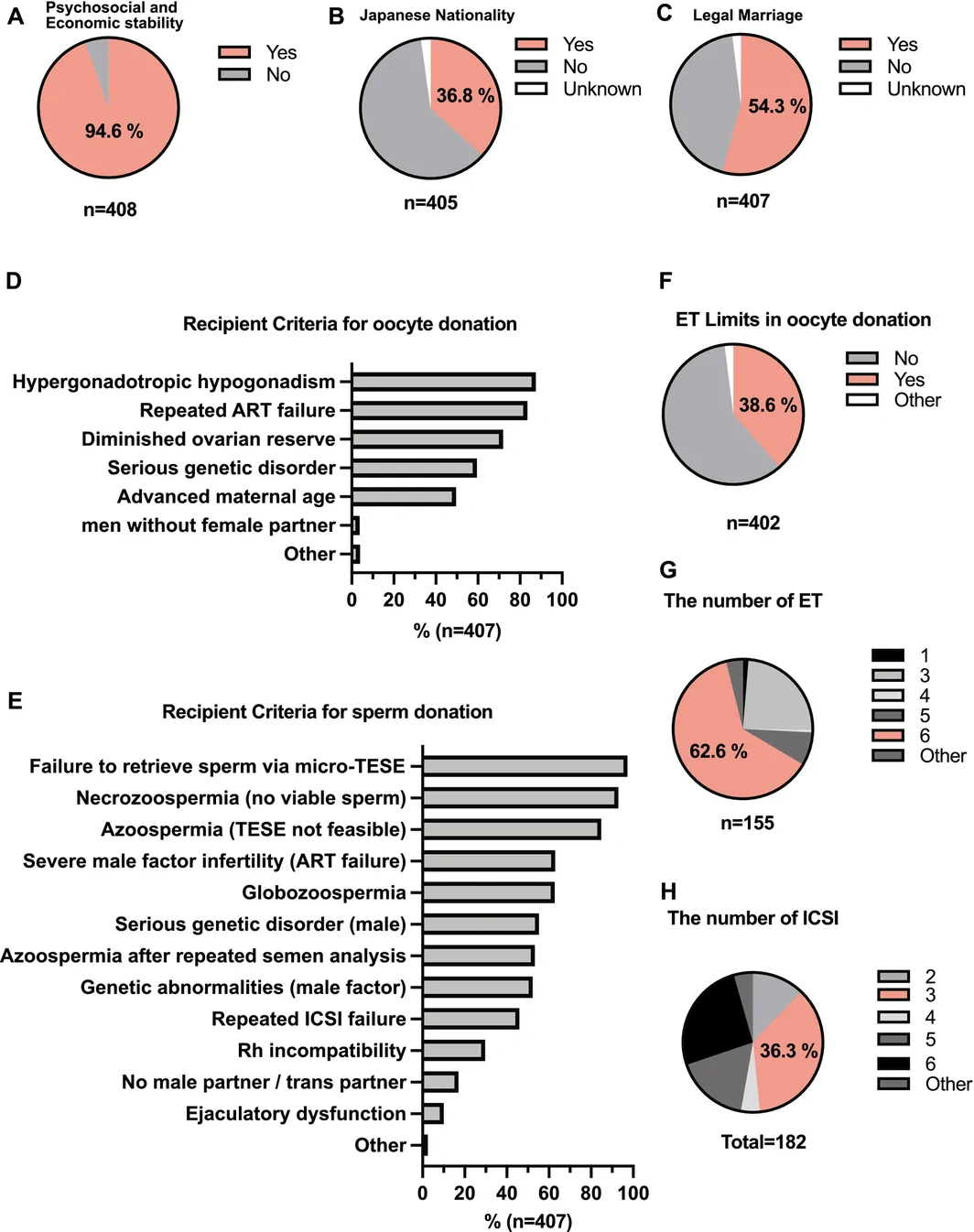
Institutional perspectives on recipient eligibility and treatment limits for third‐party ART. (A) Requirement for psychosocial and economic stability of recipient couples. (B) Requirement for Japanese nationality. (C) Requirement for legal marriage. (D) Accepted recipient indications for oocyte donation. (E) Accepted recipient indications for sperm donation. (F) Opinions on whether an upper limit should be set for embryo transfer cycles in oocyte donation. (G) Preferred upper limit for embryo transfer cycles among facilities supporting such a limit. (H) Preferred number of prior ICSI attempts before considering sperm donation for repeated ICSI failure. ART, assisted reproductive technology; ET, embryo transfer; ICSI, intracytoplasmic sperm injection; TESE, testicular sperm extraction.

For oocyte donation, the most widely accepted recipient indications were hypergonadotropic hypogonadism (356/407, 87.5%), repeated ART failure with a presumed oocyte‐related cause and very low likelihood of future pregnancy (340/407, 83.5%), diminished ovarian reserve (293/407, 72.0%), and serious genetic disease in female recipients when preimplantation/prenatal testing and pregnancy termination were not desired (242/407, 59.5%). In contrast, advanced maternal age was supported by fewer than half of the responding facilities (202/407, 49.6%), and ART use by men without a female partner was rarely supported (15/407, 3.7%) (Figure 2D). Few facilities supported limits on the number of embryo transfers in oocyte donation (155/402, 38.6%); among these, six transfers were the most commonly proposed upper limit (97/155, 62.6%) (Figure 2F,G).

The most widely accepted indications for donor sperm treatment were failure to retrieve sperm via micro‐testicular sperm extraction (TESE) (396/407, 97.3%), necrozoospermia with no viable sperm even after TESE (378/407, 92.9%), azoospermia when TESE was not feasible (345/407, 84.8%), severe male factor infertility after ART failure (256/407, 62.9%), and globozoospermia (255/407, 62.7%) (Figure 2E). Fewer than half of the responding facilities (187/407, 45.9%) supported repeated intracytoplasmic sperm injection (ICSI) failure, most commonly after three (66/182, 36.3%) or six (47/182, 25.8%) ICSI attempts (Figure 2H). Serious genetic disorders in a male partner when preimplantation/prenatal testing and pregnancy termination were not desired and chromosomal abnormalities (including structural abnormalities or Y chromosome microdeletions) were supported by more than half of the responding facilities (225/407, 55.3%; 213/407, 52.3%).

Regarding age criteria, the most common lower age limit for female recipients was 20 or 25 years across oocyte donation, donor sperm IUI, and donor sperm IVF. Upper age limits for female recipients of ART were concentrated at 43 and 45 years: oocyte donation was the most common response among those aged 45 years (119/403, 29.5%), followed by those aged 43 years (87/403, 21.6%) and 49 years (72/403, 17.9%); for donor sperm IUI, 45 years (125/400, 31.2%) and 43 years (117/400, 29.2%) were more common; and for donor sperm IVF, 43 years (129/402, 32.1%) and 45 years (118/402, 29.4%) were most common. For male partners, “no upper limit” was the most frequent response across all three treatment categories: oocyte donation (147/402, 36.6%), donor sperm IUI (139/402, 34.6%), and donor sperm IVF (139/401, 34.7%). The next most frequent upper limits were 45 and 55 years of age, with similar proportions across treatment categories (Table 3 and Table S1).

**TABLE 3:** Age limits for female recipients and male partners in third‐party ART.

	Age (years)	Female recipient		
		Oocyte donation	Donor sperm IUI	Donor sperm IVF
Lower age limit	18	87 (21.5)	92 (22.9)	93 (23.0)
	20	155 (38.4)	150 (37.4)	147 (36.4)
	25	137 (33.9)	144 (35.9)	148 (36.6)
	Other	25 (6.2)	15 (3.7)	16 (4.0)
Upper age limit	35	16 (4.0)	10 (2.5)	9 (2.2)
	37	3 (0.7)	2 (0.5)	2 (0.5)
	39	22 (5.5)	32 (8.0)	25 (6.2)
	41	10 (2.5)	20 (5.0)	17 (4.2)
	43	87 (21.6)	117 (29.2)	129 (32.1)
	45	119 (29.5)	125 (31.2)	118 (29.4)
	47	15 (3.7)	14 (3.5)	12 (3.0)
	49	72 (17.9)	36 (9.0)	43 (10.7)
	No upper limit	21 (5.2)	22 (5.5)	18 (4.5)
	Other	38 (9.4)	22 (5.5)	29 (7.2)

	Age (years)	Male partner		
		Oocyte donation	Donor sperm IUI	Donor sperm IVF
Lower age limit	18	93 (23.0)	96 (23.9)	92 (22.9)
	20	155 (38.4)	153 (38.2)	152 (37.8)
	25	134 (33.2)	133 (33.2)	138 (33.4)
	Other	22 (5.4)	19 (4.7)	20 (5.0)
Upper age limit	35	9 (2.2)	9 (2.2)	10 (2.5)
	45	103 (25.6)	120 (29.9)	116 (28.9)
	55	103 (25.6)	103 (25.6)	102 (25.4)
	No upper limit	147 (36.6)	139 (34.6)	139 (34.7)
	Other	40 (10.0)	31 (7.7)	34 (8.5)

Abbreviations: IUI, intrauterine insemination; IVF, in vitro fertilization.

### 3.5 Views on Donor Eligibility, Screening, and Donation Limits

For oocyte donors, the most common lower age limit was 20 years (163/407, 40.0%), followed by 25 years (160/407, 39.3%); the most common upper age limit was 35 years (277/408, 67.9%). For sperm donors, 20 years was also the most common lower limit for both IUI (175/404, 43.3%) and IVF (174/404, 43.1%), whereas upper age limits were more dispersed, with 40 years being the most common for sperm donation for IUI (144/404, 35.6%) and IVF (145/404, 35.9%) (Table 4).

**TABLE 4:** Age limits for Oocyte and Sperm Donor.

	Age (years)	Oocyte donor	Sperm donor for IUI	Sperm donor for IVF
Lower age limit	18	74 (18.2)	89 (22.0)	90 (22.3)
	20	163 (40.0)	175 (43.3)	174 (43.1)
	25	160 (39.3)	130 (32.2)	131 (32.4)
	Other	10 (2.5)	10 (2.5)	9 (2.2)
Upper age limit	30	38 (9.3)	28 (6.9)	28 (6.9)
	35	277 (67.9)	N/A	N/A
	40	72 (17.6)	144 (35.6)	145 (35.9)
	45	N/A	101 (25.0)	98 (24.3)
	50	N/A	41 (10.1)	40 (9.9)
	55	N/A	25 (6.2)	24 (5.9)
	60	N/A	9 (2.2)	9 (2.2)
	No upper limit	5 (1.2)	40 (9.9)	43 (10.6)
	Other	16 (3.9)	16 (4.0)	17 (4.2)

Abbreviations: IUI, intrauterine insemination; IVF, in vitro fertilization.

For oocyte donor screening, items selected by at least 60% of facilities were infectious disease testing (human immunodeficiency virus [HIV], hepatitis B virus [HBV], hepatitis C virus [HCV], and syphilis; 400/408, 98.0%), medical history (395/408, 96.8%), blood type (376/408, 92.2%), nonmedical narcotic or stimulant use (366/408, 89.7%), obstetric history (364/408, 89.2%), height and weight (358/408, 87.7%), genetic disease history (356/408, 87.3%), ovarian reserve assessment including anti‐müllerian hormone (336/408, 82.4%), transvaginal ultrasound examination (331/408, 81.1%), smoking history (328/408, 80.4%), psychiatric history (301/408, 73.8%), human T‐cell lymphotropic virus Type 1 (HTLV‐1) testing (290/408, 71.1%), chlamydia/gonorrhea screening (284/408, 69.6%), and general physical examination (278/408, 68.1%) (Figure 3A). Among facilities responding to the genetic disease screening question, 144 of 354 (40.7%) favored history taking plus selective genetic screening, 140 (39.5%) favored history taking alone, and 63 (17.8%) favored universal genetic screening (Figure 3B). Prior pregnancy or delivery was considered necessary for oocyte donors by 163 of 406 facilities (40.1%), and 314 of 407 facilities (77.1%) supported setting an upper limit for oocyte retrieval cycles. Among those supporting a limit, three retrieval cycles were the most common response (165/314, 52.5%) (Figure 3C–E).

**FIGURE 3:**
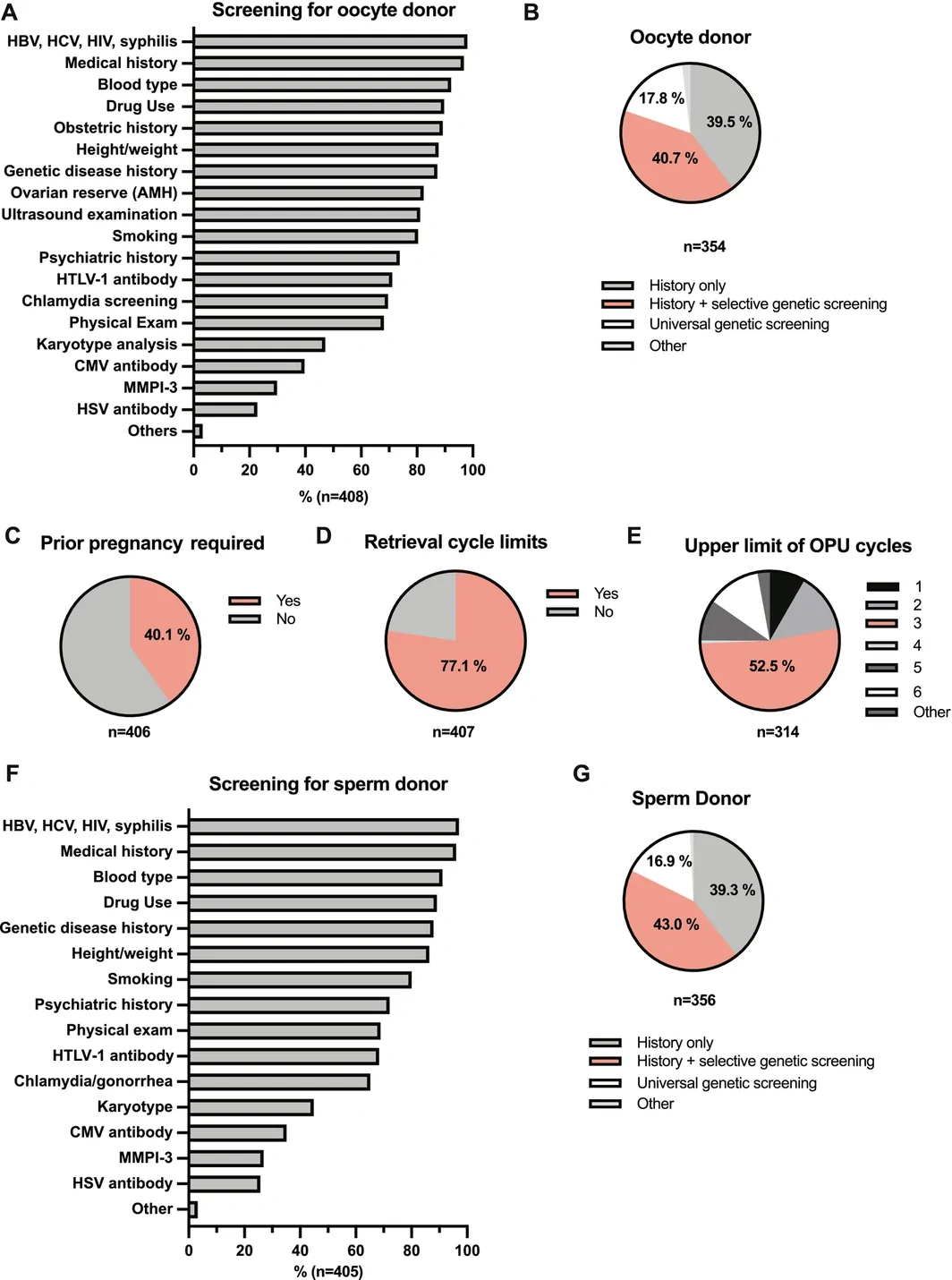
Institutional perspectives on donor screening and donation limits. (A) General screening items considered necessary for oocyte donors. (B) Preferred approach to genetic disease screening for oocyte donors. (C) Requirement for prior pregnancy or delivery history among oocyte donors. (D) Opinions on whether an upper limit should be set for oocyte retrieval cycles. (E) Preferred upper limit for oocyte retrieval cycles among facilities supporting such a limit. (F) General screening items considered necessary for sperm donors. (G) Preferred approach to genetic disease screening for sperm donors. AMH, anti‐Müllerian hormone; CMV, cytomegalovirus; HBV, hepatitis B virus; HCV, hepatitis C virus; HIV, human immunodeficiency virus; HSV, herpes simplex virus; HTLV‐1, human T‐cell lymphotropic virus Type 1; MMPI‐3, Minnesota Multiphasic Personality Inventory‐3; OPU, oocyte pickup.

For sperm donor screening, items selected by at least 60% of facilities were infectious disease testing (HIV, HBV, HCV, and syphilis; 393/405, 97.0%), medical history (389/405, 96.0%), blood type (369/405, 91.1%), nonmedical narcotic or stimulant use (361/405, 89.1%), genetic disease history (356/405, 87.9%), height and weight (350/405, 86.4%), smoking history (324/405, 80.0%), psychiatric history (292/405, 72.1%), general physical examination (279/405, 68.9%), HTLV‐1 testing (277/405, 68.4%), and chlamydia/gonorrhea screening (264/405, 65.2%) (Figure 3F). Genetic disease screening preferences were similar to those of oocyte donors: 153 of 356 facilities (43.0%) favored history taking plus selective genetic screening, 140 (39.3%) favored history taking alone, and 60 (16.9%) favored universal genetic screening (Figure 3G).

Facilities generally favor limiting the number of offspring and recipient women per donor. For the total number of offspring per donor, three offspring were the most common limit for both oocyte (111/405, 27.4%) and sperm (108/403, 26.8%) donors, followed by five offspring for sperm (99/403, 24.6%) and oocyte (92/405, 22.7%) donors. For the number of recipient women per donor, three women had the most common response for oocyte (115/403, 28.5%) and sperm (104/403, 25.8%) donors (Table 5).

**TABLE 5:** Limits on offspring and recipient numbers per donor.

Number	Total number of offspring per donor		Number of recipient women per donor	
	Oocyte donor	Sperm donor	Oocyte donor	Sperm donor
1	32 (7.9)	30 (7.4)	52 (12.9)	60 (14.9)
2	42 (10.4)	38 (9.4)	53 (13.2)	50 (12.4)
3	111 (27.4)	108 (26.8)	115 (28.5)	104 (25.8)
4	11 (2.7)	12 (3.0)	4 (1.0)	6 (1.5)
5	92 (22.7)	99 (24.6)	72 (17.9)	78 (19.4)
No upper limit	73 (18.0)	70 (17.4)	80 (19.9)	74 (18.4)
Other	44 (10.9)	46 (11.4)	27 (6.7)	31 (7.7)

### 3.6 Gamete/Embryo Management, Storage, and Compensation

Transport of third‐party gametes or embryos to another facility was considered acceptable by 204 of 405 facilities (50.4%), unacceptable by 108 (26.7%), and acceptable only under certain conditions by 93 (23.0%). The most commonly proposed storage duration was 10 years for oocytes (134/404, 33.2%), sperm (131/404, 32.4%), and embryos (136/403, 33.7%), followed by 5 years, with no upper limit for all three categories (Table 6). Preferred compensation differed according to gamete type. Using an exchange rate of 1 USD = 160.8 JPY as of June 2026, the most common responses for oocyte donation were 300,000 yen (approximately US$1,900; 110/403, 27.3%), 100,000 yen (approximately US$620; 90/403, 22.3%), and 500,000 yen (approximately US$3,100; 85/403, 21.1%), while 64 (15.9%) facilities favored no compensation. For sperm donation, the most common responses were 10,000 yen (approximately US$62; 135/403, 33.5%), 50,000 yen (approximately US$310; 102/403, 25.3%), and no compensation (78/403, 19.4%) (Table 7).

**TABLE 6:** Preferred storage duration for gametes and embryos.

Storage duration (years)	Oocyte	Sperm	Embryo
	*n* = 404	*n* = 404	*n* = 403
1	4 (1.0)	5 (1.2)	2 (0.5)
2	5 (1.2)	5 (1.2)	4 (1.0)
3	33 (8.2)	36 (8.9)	26 (6.5)
4	0 (0)	0 (0)	1 (0.2)
5	120 (29.7)	117 (29.0)	126 (31.3)
10	134 (33.2)	131 (32.4)	136 (33.7)
No upper limit	105 (26.0)	106 (26.2)	105 (26.1)
Other	3 (0.7)	4 (1.0)	3 (0.7)

**TABLE 7:** Preferred compensation for gamete donation.

Compensation (Yen)	Oocyte	Sperm
	*n* = 403	*n* = 403
None	64 (15.9)	78 (19.4)
1000	N/A	1 (0.2)
5000	N/A	22 (5.5)
10,000	3 (0.7)	135 (33.5)
50,000	17 (4.2)	102 (25.3)
100,000	90 (22.3)	40 (9.9)
300,000	110 (27.3)	N/A
500,000	85 (21.1)	N/A
Other	34 (8.4)	25 (6.2)

### 3.7 Disclosure, Telling, and Psychosocial Support

Regarding disclosure of donor information, the most common timing for providing non‐identifying donor information to recipients was adulthood of the child (108/397, 27.2%), followed by age 3 years (69/397, 17.4%), and after birth (68/397, 17.1%). For identifying donor information, adulthood was also the most common age (151/395, 38.2%), but 121 (30.6%) facilities answered that identifying information should not be disclosed at any age. For disclosure (“telling”) to a child, adulthood was the most common timing (149/404, 36.9%), followed by age 3 years (100/404, 24.8%); 29 (7.2%) facilities answered that telling should not be performed at any age (Table 8).

**TABLE 8:** Timing and extent of donor information disclosure.

Timing	Non‐identifying information of donor to recipients *n* (%)	Identifying information of donor to recipients *n* (%)	Disclosure (“telling”) to child *n* (%)
At conception	47 (11.8)	20 (5.1)	13 (3.2)
During pregnancy	14 (3.5)	2 (0.5)	4 (1.0)
After birth	68 (17.1)	29 (7.3)	36 (8.9)
At age 3 years	69 (17.4)	27 (6.8)	100 (24.8)
At adulthood	108 (27.2)	151 (38.2)	149 (36.9)
No disclosure at any age	35 (8.8)	121 (30.6)	29 (7.2)
Other	56 (14.1)	45 (11.4)	73 (18.1)

Most facilities considered psychosocial support to be necessary for both donors and recipients. For donors, psychological assessment, decision‐making, and psychological support during or after treatment were considered necessary by 393 (97.0%), 391 (96.5%), and 391 (96.5%) of 405 facilities, respectively. For the ART recipients, 399 of 405 facilities (98.5%) supported pretreatment psychological assessment addressing prior infertility‐related distress and family building reflections, 396 of 404 (98.0%) supported decision‐making support among couples and stakeholders, 397 of 405 (98.0%) supported mental health support during treatment, 398 of 406 (98.0%) supported parenting and bonding support from pregnancy to child‐rearing, and 396 of 406 (97.5%) supported disclosure‐related psychological support. Most facilities also considered repeated counseling with separate opportunities for each spouse necessary (380/402, 94.5%), and 388 of 402 facilities (96.5%) agreed that telling should be practiced in Japan.

When asked which service providers should deliver psychosocial support, facilities most often assigned donor‐focused support to coordinating organizations and donor retrieval centers, whereas recipient‐focused support was assigned most often to recipient treatment centers. For example, donor psychological assessment was attributed to coordinating organizations by 331 (84.0%) facilities, donor retrieval centers by 279 (69.2%), and recipient treatment centers by only 49 (12.2%). Conversely, recipient mental health support during treatment was attributed to recipient treatment centers by 325 (81.2%) facilities, 237 (60.2%) to coordinating organizations, and 60 (14.9%) to donor retrieval centers (Table 9).

**TABLE 9:** Provision of psychosocial support across different service providers in third‐party ART.

Support item	Coordinating organizations *n* (%)	Donor retrieval centers *n* (%)	Recipient treatment centers *n* (%)
Donor psychological assessment	331 (84.0)	279 (69.2)	49 (12.2)
Donor decision‐making support	333 (84.5)	289 (71.7)	51 (12.8)
Donor support during/after treatment	303 (76.9)	314 (77.9)	50 (12.5)
Care for prior infertility‐related distress (recipients)	261 (66.2)	59 (14.6)	271 (67.8)
Support for reflection on treatment and family building (recipients)	283 (71.8)	55 (13.6)	273 (68.2)
Psychological assessment (recipients)	290 (73.6)	54 (13.4)	295 (73.8)
Decision‐making support (recipients and stakeholders)	287 (72.8)	59 (14.6)	311 (77.8)
Mental health support during treatment (recipients)	237 (60.2)	60 (14.9)	325 (81.2)
Parenting support (posttreatment to child‐rearing)	231 (58.6)	42 (10.4)	217 (54.2)
Disclosure‐related psychological support (family formation)	252 (64.0)	44 (10.9)	208 (52.0)

## 4 Discussion

This nationwide survey clarified institutional perspectives regarding third‐party ART among JSOG‐registered ART facilities in Japan. The response rate was relatively high, suggesting that our findings provide useful insights into the views of Japanese ART providers. Additionally, more than half of the responding facilities were clinics without beds, suggesting that the respondent profile reflects the current clinic‐based structure of ART practice in Japan to some extent. However, only 22 (5.3%) of responding facilities reported experience with third‐party ART. As this proportion was based on responding facilities and the practice status of nonresponding facilities is unknown, it may not fully represent the national prevalence of third‐party ART; nevertheless, it suggests that clinical implementation was limited among participating facilities despite potential patient demand. For international context, the International Federation of Fertility Societies (IFFS) Triennial Report 2025 indicates that sperm and oocyte donation are widely practiced internationally, with both reported as commonly used in approximately two‐thirds of responding countries [8]. However, the IFFS findings reflect country‐level reports, whereas our survey assessed facility‐level experience and perspectives in Japan; these findings therefore provide context rather than a direct quantitative comparison. The IFFS report also shows that third‐party reproduction in many countries is governed primarily by national legislation, whereas sperm donation in Japan is regulated through professional organization standards and guidelines [8]. These international comparisons highlight the contrast between the limited implementation and professional‐society‐based governance of third‐party reproduction in Japan and the broader availability and national regulatory frameworks observed in many other countries. The IFFS report also identifies access to services unavailable in the country of residence, lower costs, and perceived higher quality of care as reported motivations for cross‐border reproductive care [8]. In Japan, previous research has documented obstetric care following oocyte donation performed overseas [6]. These findings highlight the relevance of cross‐border reproductive care to discussions of third‐party reproduction in Japan, although our survey did not assess patient travel or its underlying reasons. The high proportion of facilities reporting counseling systems also suggests that many ART centers already recognize the need for structured decision support, even when they do not currently provide third‐party ART.

Findings on recipient eligibility indicate that Japanese ART facilities tend to emphasize the psychosocial and economic stability of recipient couples. This likely reflects the recognition that third‐party reproduction is not only a medical intervention but also a family building process involving the long‐term welfare of donor‐conceived children, recipients, and donors. Opinions were divided regarding Japanese nationality and legal marriage as eligibility requirements. In Japan, ART is not limited to legally married couples under the current JSOG guidelines [9]. Eligibility criteria for third‐party reproduction vary among countries, particularly with regard to marital status and nationality. In the United Kingdom, donor gametes can be used by heterosexual couples, same‐sex couples, and single women within a regulatory framework that emphasizes consent, legal parenthood, and child welfare [10]. Similarly, in the United States, there is no nationwide requirement that recipients be legally married, and the American Society for Reproductive Medicine (ASRM) Ethics Committee states that access to fertility treatment should not be restricted based on marital status, sexual orientation, or gender identity [11]. In contrast, Taiwan represents a more restrictive model, as the current Assisted Reproduction Act defines a recipient couple as a husband and wife [12]. Future Japanese guidelines should carefully consider whether formal requirements, such as legal marital status and nationality, are necessary and proportionate while balancing the welfare of donor‐conceived children, recipients' reproductive autonomy, legal stability of parenthood, and equitable access to treatment.

Regarding the indications for female recipients, conditions such as hypergonadotropic hypogonadism, diminished ovarian reserve, serious hereditary disease, and unsuccessful ART using autologous oocytes are widely supported, and these are broadly consistent with the indications for oocyte donation described by the ASRM [2]. In contrast, only approximately half of the facilities supported advanced maternal age as an indication for oocyte donation, suggesting a cautious attitude among Japanese ART facilities toward proceeding to third‐party oocyte donation based on age alone. Although the risk of embryonic chromosomal abnormalities in oocyte donation depends primarily on donor age, maternal and perinatal risks, including hypertensive disorders of pregnancy, preterm birth, and low birth weight, as well as long‐term capacity for childrearing, are also closely related to recipient age [13, 14, 15, 16]. Therefore, decisions regarding oocyte donation require a comprehensive assessment that includes not only medical efficacy but also maternal safety, child welfare, and psychosocial evaluations. For male recipients, indications such as azoospermia, failure to retrieve sperm via micro‐TESE, severe sperm abnormalities, repeated fertilization failure after ICSI, and risk of transmitting hereditary disease were supported, consistent with the ASRM indications for donor sperm use and the ethical framework for third‐party reproduction described by the European Society of Human Reproduction and Embryology (ESHRE) [2, 17]. Taken together, these findings suggest that Japanese ART facilities regard third‐party gamete donation as a relatively acceptable option when medical necessity is clearly defined, while showing caution toward expanding indications based on age alone.

Age and treatment limitations were also important factors in this survey. The upper age limits for female recipients were concentrated at 43–45 years, whereas many facilities reported no upper age limits for male partners. For female recipients, this pattern likely reflects concerns about obstetric risk. Systematic reviews have shown that oocyte donation pregnancies are associated with increased risks of hypertensive disorders of pregnancy, pre‐eclampsia, preterm birth, and low birth weight compared with autologous IVF pregnancies or spontaneous conception [13, 14, 15]. Additionally, under the Japanese public health insurance system for ART, treatment is covered for up to six cycles when a woman is < 40 years at the start of treatment and up to three cycles when she is aged 40–43 years; women aged ≥ 43 years are not eligible for insurance coverage [18]. This policy framework may partly explain why many facilities selected an upper age limit of approximately 43 years and preferred treatment limits of three or six ET cycles. Although many facilities did not support an upper age limit for male partners, a substantial proportion selected limits of 45 or 55 years, suggesting that paternal age may also be considered from the perspectives of childrearing capacity and child welfare. Therefore, recipient age criteria and limits on ET cycles should be discussed not only in terms of treatment efficacy but also in terms of maternal‐fetal safety and long‐term family welfare.

Donor‐age limits also require careful consideration. Many facilities support a lower age limit of 18–20 years, suggesting a general view that gamete donors should be legal adults. However, a substantial proportion selected 25 years as the lower limit, which may reflect the view that donors should be sufficiently mature adults who can understand the medical risks of donation, the possibility of future access to donor information by donor‐conceived offspring, and long‐term responsibilities associated with family building. The ASRM recommends that sperm donors should be of legal adult age and be ideally at least 21 years old, and that oocyte donors should be of legal age and preferably 21–34 years old [2]. In the United Kingdom, the current professional guidance generally sets donor age ranges at 18–45 years for sperm donors and 18–35 years for oocyte donors, whereas European data show that donor age limits vary across jurisdictions [1, 19]. The upper age limits appear to be driven mainly by medical considerations. For oocytes, increasing age is associated with a decline in oocyte quality and increased embryonic aneuploidy, supporting an upper age limit of approximately the mid‐30s for oocyte donors [2, 20]. Although sperm production continues throughout life, advanced paternal age has been associated with changes in semen parameters, sperm DNA damage, de novo mutations, and increased risks of neurodevelopmental and psychiatric disorders in offspring, providing a medical rationale for upper age limits for sperm donors [16, 21]. However, overly strict age restrictions may reduce donor availability and limit access to treatments and donor diversity. Therefore, future Japanese guidelines should define donor age criteria by balancing the different age‐related biological effects on oocytes and sperm with donor maturity, medical safety, welfare of donor‐conceived children, and the practical need to secure an adequate donor pool.

Only approximately 40% of facilities considered a history of pregnancy or delivery necessary. The ASRM states that proven fertility in a donor is desirable but not required, and our findings similarly suggest caution against treating prior pregnancy or delivery as an absolute eligibility criterion for oocyte donation [2]. However, in Japan, the JISART guidelines have historically described oocyte donors as adults with children [22]. This requirement may reflect not only the medical value of demonstrating reproductive potential but also a donor‐protective rationale because the possibility of complications from oocyte retrieval or future effects on the donor's own fertility cannot be completely excluded; requiring prior childbirth may help reduce the risk of later regret, psychological burden, or disputes if the donor is subsequently unable to have children. Thus, whether prior pregnancy or delivery is required must be discussed not only as a marker of fertility but also in relation to the donor's future reproductive autonomy, physical risk, psychological burden, and donor availability. In contrast, approximately 80% of facilities supported setting an upper limit on oocyte retrieval cycles, with three cycles being the most commonly selected limit. The ASRM notes that available data do not allow a definitive maximum number of donations to be established, but considers it reasonable and prudent to limit stimulated cycles for an individual oocyte donor to no more than six, given the potential cumulative physical and psychological burdens, as well as risks such as ovarian hyperstimulation syndrome and acute complications related to oocyte retrieval [23]. Therefore, the preference for a three‐cycle limit in this survey may reflect a more conservative, donor‐protective attitude among Japanese ART facilities.

Regarding donor eligibility, most facilities supported screening for infectious diseases, medical history, lifestyle factors, obstetric history, and a family history of genetic disorders. These priorities are broadly consistent with international guidelines recommending medical, infectious disease, genetic, and psychological evaluations of gamete and embryo donors [2]. In particular, genetic screening should be discussed not only as a donor‐exclusion tool but also as part of preconception care. In recent years, carrier screening, especially expanded carrier screening, has been increasingly used internationally before pregnancy or fertility treatment to help individuals and couples understand the genetic risks of having offspring and consider available reproductive options [24, 25]. The ASRM notes that expanded carrier screening may be appropriate for gamete donation and emphasizes the importance of genetic counseling regarding residual risk and reproductive implications [2]. The ACOG similarly considers ethnicity‐specific, pan‐ethnic, and expanded carrier screening to be acceptable strategies in prepregnancy and prenatal care, while stressing that testing should be voluntary and accompanied by appropriate counseling [25]. In this survey, many facilities preferred history‐taking with selective genetic testing rather than universal expanded carrier screening. This cautious attitude may reflect concerns that broader testing could increase donor exclusion, generate incidental or difficult‐to‐interpret findings, and require specialized genetic counseling [24]. Therefore, if carrier screening is incorporated into third‐party gamete donation practices in Japan, the scope of tested conditions, criteria for donor exclusion, explanation of residual risk, handling of variants, and access to genetic counseling should be standardized.

Japanese ART facilities support relatively cautious operational rules regarding donor‐use limits, storage duration for gametes and embryos, and donor compensation. For both oocyte and sperm donors, the most commonly preferred limit was three offspring or three recipient women per donor, which is stricter than the current JSOG view on artificial insemination by donor, limiting offspring from the same sperm donor to no more than 10, and more conservative than many European regulations [26]. Notably, the 2026 JSOG draft revision proposes limiting donor sperm use to no more than 10 offspring or five families from the same donor, suggesting a possible shift toward management based not only on offspring number but also on family number [27]. In the 2024 ESHRE/European IVF Monitoring Consortium (EIM) survey, limits on the number of offspring from the same donor were reported in 34 European countries for sperm donation and 26 countries for oocyte donation, ranging between 1 and 10 offspring; some countries used limits based on the number of families or recipient women. Limits on the number of oocyte donations have been reported in 12 countries, most commonly in 4–6 donations [1]. Regarding storage duration, 5 or 10 years was commonly selected in the present survey, which is broadly consistent with the ESHRE/EIM finding that 10 years was the most frequent legal maximum for embryo cryopreservation in Europe. However, approximately one‐quarter of facilities supported no upper limit, suggesting a desire for flexible operation if consent renewal, withdrawal, posthumous use, disposition of unused embryos, and long‐term information management are clearly regulated [1]. Compensation preferences differed by gamete type, with higher amounts favoring oocyte donation than sperm donation, likely reflecting the greater physical burden, inconvenience, and risk of complications associated with ovarian stimulation and oocyte retrieval. This pattern is also consistent with European practice, where reimbursement or compensation is available for sperm donors in 32 countries and oocyte donors in 31 countries. This pattern is also consistent with the IFFS Triennial Report 2025, in which reimbursement for time and expenses was the most reported form of compensation for both sperm and oocyte donation, whereas compensation beyond reimbursement was less common [8]. Countries with defined maximum amounts generally set higher compensation for oocyte donors than for sperm donors [1]. Therefore, future Japanese guidelines should link donor‐use limits, storage duration, and compensation principles to a national registry and consent management framework while balancing donor protection, donor availability, treatment access, reduction of consanguinity risk, and welfare of donor‐conceived children.

Most of the facilities recognized the need for disclosure‐related support and psychosocial care for donors, recipients, and donor‐conceived children. Notably, although there was broad support for telling children about their donor conception, support for telling at an early age was relatively limited, with a substantial proportion of respondents favoring telling in adulthood. In the Japanese context, this finding may suggest that acceptance of telling itself has advanced further than consensus regarding when and how disclosure should begin. This finding should also be considered in the context of an international shift away from the traditional emphasis on secrecy and donor anonymity. The IFFS Triennial Report 2025 describes a trend toward increasing the amount of donor‐related information made available to offspring, recipients, and donors [8]. This distinction is important because disclosure is increasingly understood not as a single event at a particular age, but as an ongoing developmental process in which information is introduced gradually in an age‐appropriate manner [28]. Recent studies suggest that early and age‐appropriate disclosure is not associated with adverse psychological adjustment in donor‐conceived families [29], although family experiences are heterogeneous and some parents, particularly fathers in donor insemination families, may require continuing support over time [30]. Therefore, in Japan, counseling systems should support recipients in treatment decision‐making through pregnancy, parenting, and child disclosure, including guidance on how to initiate and continue age‐appropriate discussions from an early stage, while also providing independent opportunities for donors and each member of a recipient couple to discuss concerns.

As an exploratory analysis, we compared questionnaire responses between facilities with and without experience providing ART using third‐party gametes. Given the small number of facilities with such experience (*n* = 22), the findings should be interpreted with caution. Facilities with reported experience had higher annual ART volumes and more embryologists, whereas no differences in questionnaire responses concerning institutional perspectives remained statistically significant after multiple‐comparison adjustment. These exploratory findings should be interpreted cautiously because only 22 facilities reported experience. The absence of statistically significant differences does not establish equivalence between the groups.

This study has several limitations. The responses were self‐reported by facility representatives and may not fully reflect actual clinical practice or the diversity of opinions within each institution. Additionally, because few facilities have direct experience with third‐party ART, some responses may represent hypothetical views rather than practice‐based judgments. The survey also focused on ART facilities and did not directly capture the perspectives of donors, recipients, donor‐conceived offspring, perinatal care providers, or policymakers. Nevertheless, this study provides important nationwide evidence regarding the issues that Japanese ART facilities consider relevant for future implementation. Establishing national guidelines and a registry system that integrates medical safety, donor protection, recipient support, information disclosure, and the rights and welfare of donor‐conceived children are warranted.

## 5 Conclusion

This nationwide survey provided the first comprehensive overview on the perspectives of Japanese ART facilities using third‐party gametes. Although clinical experience with third‐party ART in Japan remains limited, Japanese ART facilities showed broad agreement on the importance of eligibility criteria, counseling, psychosocial support, and welfare of donor‐conceived children, while opinions varied regarding specific operational criteria. These findings support the development of national guidelines and a registry system that balances medical safety, donor protection, recipient autonomy, equitable access, and the rights and welfare of donor‐conceived children.

## Funding

This work was supported by the Children and Families Agency, 25DB0201. Ministry of Health, Labour and Welfare, 25FB2001. Japan Society for the Promotion of Science, 25K20078.

## Conflicts of Interest

The authors declare no conflicts of interest.

## Supporting information


**Table S1:** Exploratory comparisons of questionnaire responses by facility type.

**Table S2:** Exploratory comparisons of questionnaire responses by experience with third‐party reproduction.

**Data S1:** Questionnaire for ART facilities.

## Data Availability

The data that support the findings of this study are available from the corresponding author upon reasonable request.
