# Supplementary material for: Institutional Perspectives on Assisted Reproductive Technology Using Third‐Party Gametes in Japan: A Nationwide Survey of Assisted Reproductive Technology Facilities
Source: Reprod Med Biol. 2026 Nov 3;25(1):e70108. doi: 10.1002/rmb2.70108 (PMC13732116; doi:10.1002/rmb2.70108)
Supplement: Supplementary file 1 [file RMB2-25-e70108-s003.docx]

**Supplementary Material. Questionnaire for ART facilities**

**Section 1. Facility Characteristics**

Please provide basic information about your facility.

**1.** Location of your facility (please enter the prefecture):

**Prefecture:** ________________

**2.** Name of your facility:

**Facility name:** ________________

**3.** Name of the person responsible for your facility:

**Name:** ________________

**4.** Type of facility:

**Options:** University hospital / General hospital / Clinic with inpatient beds / Clinic without inpatient beds

**5.** Annual number of ART cycles at your facility (oocyte retrieval cycles plus frozen-thawed embryo transfer cycles; number in 2024):

**Number of cycles:** ________________ cycles

**6.** Number of obstetricians and gynecologists engaged in reproductive medicine at your facility:

**Number:** ________________ physicians

**7.** Number of board-certified reproductive medicine specialists at your facility:

**Number:** ________________ physicians

**8.** Number of embryologists at your facility:

**Number:** ________________ embryologists

**9.** Does your facility provide counseling for purposes such as medical information provision, medical explanation, and psychosocial support in reproductive medicine?

**Options:** Yes / No

**10.** If you answered Yes to Question 9, please select all providers of counseling. If an applicable profession is not listed, please enter it under Other:

**Options:** Physician / Reproductive medicine specialist / Nurse / Certified nurse in reproductive nursing / Clinical psychologist / Certified public psychologist / Reproductive psychology counselor / Infertility counselor / Social worker / Other: ____________

**11.** If you answered Yes to Question 9, please select all items included in reproductive medicine counseling at your facility. If an applicable item is not listed, please enter the content under Other:

**Options:** Interview for the purpose of providing medical information / Coordination with health care services and welfare services / Interview for the purpose of psychosocial support / Other: ____________

**Section 2. Implementation Status of ART Using Third-Party Donated Gametes**

Please answer regarding the implementation status at your facility of assisted reproductive technology using third-party donated gametes (artificial insemination, in vitro fertilization, and intracytoplasmic sperm injection). Facilities that have not implemented these procedures should answer only Questions 1, 12, and 23. In this questionnaire, a gamete provider is referred to as a donor, a woman who undergoes ART using donated gametes is referred to as a female recipient, and her spouse is referred to as the male partner.

**1.** Has your facility ever performed in vitro fertilization or intracytoplasmic sperm injection using third-party donated oocytes?

**Options:** Yes / No

**2.** Number of cases of in vitro fertilization (or intracytoplasmic sperm injection) using third-party donated oocytes at your facility:

**Response:** ________________ cases

**3.** Minimum age of oocyte donors:

**Response:** ________________ years

**4.** Maximum age of oocyte donors:

**Response:** ________________ years

**5.** Marital status of oocyte donors:

**Options:** Married / Unmarried / Both experienced

**6.** Minimum age of spouses of oocyte donors:

**Response:** ________________ years

**7.** Maximum age of spouses of oocyte donors:

**Response:** ________________ years

**8.** Minimum age of female recipients undergoing IVF using donated oocytes:

**Response:** ________________ years

**9.** Maximum age of female recipients undergoing IVF using donated oocytes:

**Response:** ________________ years

**10.** Minimum age of male partners in IVF using donated oocytes:

**Response:** ________________ years

**11.** Maximum age of male partners in IVF using donated oocytes:

**Response:** ________________ years

**12.** Has your facility ever performed artificial insemination using third-party donated sperm?

**Options:** Yes / No

**13.** Number of cases of artificial insemination using third-party donated sperm at your facility:

**Response:** ________________ cases

**14.** Minimum age of sperm donors for artificial insemination at your facility:

**Response:** ________________ years

**15.** Maximum age of sperm donors for artificial insemination at your facility:

**Response:** ________________ years

**16.** Marital status of sperm donors for artificial insemination:

**Options:** Married / Unmarried / Both experienced

**17.** Minimum age of spouses of sperm donors for artificial insemination:

**Response:** ________________ years

**18.** Maximum age of spouses of sperm donors for artificial insemination:

**Response:** ________________ years

**19.** Minimum age of female recipients undergoing artificial insemination using donated sperm:

**Response:** ________________ years

**20.** Maximum age of female recipients undergoing artificial insemination using donated sperm:

**Response:** ________________ years

**21.** Minimum age of male partners in artificial insemination using donated sperm:

**Response:** ________________ years

**22.** Maximum age of male partners in artificial insemination using donated sperm:

**Response:** ________________ years

**23.** Has your facility ever performed in vitro fertilization using third-party donated sperm?

**Options:** Yes / No

**24.** Number of cases of in vitro fertilization using third-party donated sperm at your facility:

**Response:** ________________ cases

**25.** Minimum age of sperm donors for in vitro fertilization at your facility:

**Response:** ________________ years

**26.** Maximum age of sperm donors for in vitro fertilization at your facility:

**Response:** ________________ years

**27.** Marital status of sperm donors for in vitro fertilization:

**Options:** Married / Unmarried / Both experienced

**28.** Minimum age of spouses of sperm donors for in vitro fertilization:

**Response:** ________________ years

**29.** Maximum age of spouses of sperm donors for in vitro fertilization:

**Response:** ________________ years

**30.** Minimum age of female recipients undergoing IVF using donated sperm:

**Response:** ________________ years

**31.** Maximum age of female recipients undergoing IVF using donated sperm:

**Response:** ________________ years

**32.** Minimum age of male partners in IVF using donated sperm:

**Response:** ________________ years

**33.** Maximum age of male partners in IVF using donated sperm:

**Response:** ________________ years

**Section 3. Opinions Regarding Recipients and Male Partners**

If assisted reproductive technology using third-party donated gametes (artificial insemination or in vitro fertilization) were to be performed, please answer what you consider appropriate regarding female recipients and their male partners.

**1.** Do you think that the female recipient and her male partner should be a couple who are socially recognized as able to raise the child to be born in a stable manner, considering factors such as the couple's health status, psychological stability, and economic situation?

**Options:** Yes / No

**2.** Do you think that the female recipient and her male partner should be limited to persons who have Japanese nationality?

**Options:** Yes / No

**3.** Do you think that the female recipient and her male partner should be limited to a legally married couple?

**Options:** Yes / No

**4.** Please select all conditions that you consider appropriate for female recipients undergoing IVF using donated oocytes. If an applicable condition is not listed, please enter it under Other:

**Options:** Women with hypergonadotropic hypogonadism / Women of advanced maternal age / Women with diminished ovarian reserve / Cases in which the wife is a carrier of, or affected by, a serious hereditary disease and does not wish to undergo preimplantation or prenatal testing or pregnancy termination / Cases in which pregnancy or childbirth has not been achieved even with ART using the couple's own gametes, the cause is judged to be related to oocytes, and a physician determines that the possibility of future pregnancy is extremely low / Men without a female partner / Other: ____________

**5.** Do you think that an upper limit should be set for the number of embryo transfers performed for female recipients undergoing IVF using donated oocytes?

**Options:** Yes / No

**6.** If you answered Yes to Question 5, please select one number that you consider appropriate as the upper limit. If an applicable number is not listed, please enter it under Other:

**Options:** 1 time / 2 times / 3 times / 4 times / 5 times / 6 times / Other: ____ times

**7.** Please select one age that you consider appropriate as the lower age limit for female recipients undergoing IVF using donated oocytes. If an applicable age is not listed, please enter it under Other:

**Options:** 18 years / 20 years / 25 years / Other: ____ years

**8.** Please select one age that you consider appropriate as the upper age limit for female recipients undergoing IVF using donated oocytes. If an applicable age is not listed, please enter it under Other:

**Options:** 35 years / 37 years / 39 years / 41 years / 43 years / 45 years / 47 years / 49 years / No upper limit / Other: ____ years

**9.** Please select one age that you consider appropriate as the lower age limit for male partners in IVF using donated oocytes. If an applicable age is not listed, please enter it under Other:

**Options:** 18 years / 20 years / 25 years / Other: ____ years

**10.** Please select one age that you consider appropriate as the upper age limit for male partners in IVF using donated oocytes. If an applicable age is not listed, please enter it under Other:

**Options:** 35 years / 45 years / 55 years / No upper limit / Other: ____ years

**11.** Please select all conditions that you consider appropriate for male partners in assisted reproductive technology using donated sperm (artificial insemination or in vitro fertilization). If an applicable condition is not listed, please enter it under Other:

**Options:** Azoospermia confirmed by multiple semen analyses / Azoospermia with no sperm found even using microTESE / Azoospermia with a physical reason that prevents TESE from being performed / Diagnosis of necrozoospermia, with no viable sperm found even using TESE / Diagnosis of globozoospermia, a type of teratozoospermia / No fertilization despite multiple attempts at intracytoplasmic sperm injection (ICSI) / Cases in which pregnancy or childbirth has not been achieved even with ART between partners, the cause is judged to be related to sperm, and a physician determines that the possibility of future pregnancy is extremely low / Cases in which the cause of the husband's abnormal semen findings is a chromosomal abnormality, such as a structural abnormality or Y-chromosome microdeletion, and transmission to the child is a concern / Cases in which the husband is a carrier of, or affected by, a serious hereditary disease and does not wish to undergo preimplantation or prenatal testing or pregnancy termination / Cases in which the wife is Rh-negative and has severe Rh sensitization, and the husband is Rh-positive / Cases in which the husband has ejaculatory dysfunction / Women without a male partner, or women with a transgender male partner / Other: ____________

**12.** If you selected item 6 in Question 11, please select one number that you consider appropriate as the lower limit for the number of previous ICSI attempts in ART between partners as a condition for the male partner in IVF using donated sperm. If an applicable number is not listed, please enter it under Other:

**Options:** 1 time / 2 times / 3 times / 4 times / 5 times / 6 times / Other: ____ times

**13.** Please select one age that you consider appropriate as the female recipients undergoing artificial insemination using donated sperm - lower age limit. If an applicable age is not listed, please enter it under Other:

**Options:** 18 years / 20 years / 25 years / Other: ____ years

**14.** Please select one age that you consider appropriate as the female recipients undergoing artificial insemination using donated sperm - upper age limit. If an applicable age is not listed, please enter it under Other:

**Options:** 35 years / 37 years / 39 years / 41 years / 43 years / 45 years / 47 years / 49 years / No upper limit / Other: ____ years

**15.** Please select one age that you consider appropriate as the male partners in artificial insemination using donated sperm - lower age limit. If an applicable age is not listed, please enter it under Other:

**Options:** 18 years / 20 years / 25 years / Other: ____ years

**16.** Please select one age that you consider appropriate as the male partners in artificial insemination using donated sperm - upper age limit. If an applicable age is not listed, please enter it under Other:

**Options:** 35 years / 45 years / 55 years / No upper limit / Other: ____ years

**17.** Please select one age that you consider appropriate as the female recipients undergoing IVF using donated sperm - lower age limit. If an applicable age is not listed, please enter it under Other:

**Options:** 18 years / 20 years / 25 years / Other: ____ years

**18.** Please select one age that you consider appropriate as the female recipients undergoing IVF using donated sperm - upper age limit. If an applicable age is not listed, please enter it under Other:

**Options:** 35 years / 37 years / 39 years / 41 years / 43 years / 45 years / 47 years / 49 years / No upper limit / Other: ____ years

**19.** Please select one age that you consider appropriate as the male partners in IVF using donated sperm - lower age limit. If an applicable age is not listed, please enter it under Other:

**Options:** 18 years / 20 years / 25 years / Other: ____ years

**20.** Please select one age that you consider appropriate as the male partners in IVF using donated sperm - upper age limit. If an applicable age is not listed, please enter it under Other:

**Options:** 35 years / 45 years / 55 years / No upper limit / Other: ____ years

**Section 4. Opinions Regarding Donors**

If assisted reproductive technology using third-party donated gametes (artificial insemination or in vitro fertilization) were to be performed, please answer what you consider appropriate regarding donors.

**1.** Please select all items that you consider necessary as screening tests or information to be obtained for oocyte donors. If an applicable item is not listed, please enter it under Other:

**Options:** Height and weight / Pregnancy and childbirth history / Smoking history / Medical history / History of non-medical narcotic or stimulant use / General physical examination, such as auscultation and palpation / Blood type (ABO, Rh) / Common sexually transmitted infections (HIV, HBV, HCV, syphilis) / HTLV-1 testing / CMV testing / Chlamydia and gonorrhea testing / Herpes simplex virus testing / Transvaginal ultrasonography / Ovarian reserve assessment / Presence or absence of hereditary disease / Chromosomal testing / Presence or absence of mental disorders / Psychological testing, such as MMPI-3 / Other: ____________

**2.** If you selected item 15, Presence or absence of hereditary disease, in Question 1, please select one method that you consider appropriate when screening oocyte donors for hereditary diseases. If an applicable method is not listed, please enter it under Other:

**Options:** Medical interview only, for example asking whether the donor or blood relatives within the third degree have a hereditary disease / Medical interview, with genetic screening testing as needed, such as BRCA genes, cystic fibrosis, thalassemia, and fragile X syndrome / Genetic screening testing for all donors, such as BRCA genes, cystic fibrosis, thalassemia, and fragile X syndrome / Other: ____________

**3.** Do you think that a history of pregnancy or childbirth should be required as a condition for oocyte donors?

**Options:** Yes / No

**4.** Do you think that an upper limit should be set for the number of oocyte retrieval procedures performed for oocyte donors?

**Options:** Yes / No

**5.** If you answered Yes to Question 4, please select one number that you consider appropriate as the upper limit for the number of oocyte retrieval procedures for oocyte donors. If an applicable number is not listed, please enter it under Other:

**Options:** 1 time / 2 times / 3 times / 4 times / 5 times / 6 times / Other: ____ times

**6.** Please select one age that you consider appropriate as the lower age limit for oocyte donors. If an applicable age is not listed, please enter it under Other:

**Options:** 18 years / 20 years / 25 years / Other: ____ years

**7.** Please select one age that you consider appropriate as the upper age limit for oocyte donors. If an applicable age is not listed, please enter it under Other:

**Options:** 30 years / 35 years / 40 years / No upper limit / Other: ____ years

**8.** Please select one number that you consider appropriate as the upper limit for the total number of children born from oocytes donated by the same person. If an applicable number is not listed, please enter it under Other:

**Options:** 1 child / 2 children / 3 children / 4 children / 5 children / No upper limit / Other: ____ children

**9.** Please select one number that you consider appropriate as the upper limit for the number of women who may have children from oocytes donated by the same person. If an applicable number is not listed, please enter it under Other:

**Options:** 1 woman / 2 women / 3 women / 4 women / 5 women / No upper limit / Other: ____ women

**10.** Please select all items that you consider necessary as screening tests or information to be obtained for sperm donors. If an applicable item is not listed, please enter it under Other:

**Options:** Height and weight / Smoking history / Medical history / History of non-medical narcotic or stimulant use / General physical examination, such as auscultation and palpation / Blood type (ABO, Rh) / Common sexually transmitted infections (HIV, HBV, HCV, syphilis) / HTLV-1 testing / CMV testing / Chlamydia and gonorrhea testing / Herpes simplex virus testing / Presence or absence of hereditary disease / Chromosomal testing / Presence or absence of mental disorders / Psychological testing, such as MMPI-3 / Other: ____________

**11.** If you selected item 12, Presence or absence of hereditary disease, in Question 10, please select one method that you consider appropriate when screening sperm donors for hereditary diseases. If an applicable method is not listed, please enter it under Other:

**Options:** Medical interview only, for example asking whether the donor or blood relatives within the second degree have a hereditary disease / Medical interview, with genetic screening testing as needed, such as Y-chromosome microdeletions, cystic fibrosis, thalassemia, and fragile X syndrome / Genetic screening testing for all donors, such as Y-chromosome microdeletions, cystic fibrosis, thalassemia, and fragile X syndrome / Other: ____________

**12.** Please select one age that you consider appropriate as the lower age limit for sperm donors used for artificial insemination. If an applicable age is not listed, please enter it under Other:

**Options:** 18 years / 20 years / 25 years / Other: ____ years

**13.** Please select one age that you consider appropriate as the upper age limit for sperm donors used for artificial insemination. If an applicable age is not listed, please enter it under Other:

**Options:** 30 years / 40 years / 45 years / 50 years / 55 years / 60 years / No upper limit / Other: ____ years

**14.** Please select one age that you consider appropriate as the lower age limit for sperm donors used for IVF. If an applicable age is not listed, please enter it under Other:

**Options:** 18 years / 20 years / 25 years / Other: ____ years

**15.** Please select one age that you consider appropriate as the upper age limit for sperm donors used for IVF. If an applicable age is not listed, please enter it under Other:

**Options:** 30 years / 40 years / 45 years / 50 years / 55 years / 60 years / No upper limit / Other: ____ years

**16.** Please select one number that you consider appropriate as the upper limit for the total number of children born from sperm donated by the same person. If an applicable number is not listed, please enter it under Other:

**Options:** 1 child / 2 children / 3 children / 4 children / 5 children / No limit / Other: ____ children

**17.** When sperm is provided to multiple women, please select one number that you consider appropriate as the upper limit for the number of women who may have children from sperm donated by the same person. If an applicable number is not listed, please enter it under Other:

**Options:** 1 woman / 2 women / 3 women / 4 women / 5 women / No limit / Other: ____ women

**Section 5. Opinions Regarding the Handling of Third-Party Donated Gametes**

If assisted reproductive technology using third-party donated gametes were to be performed, please answer what you consider appropriate regarding the handling of frozen gametes.

**1.** Do you think it is acceptable to transport third-party donated sperm or oocytes, and embryos obtained from them, to another facility?

**Options:** Yes / No / Acceptable under certain conditions

**2.** If you answered Acceptable under certain conditions in Question 1, please describe the specific conditions:

**Conditions:** ________________

**3.** Please select one period that you consider appropriate as the maximum storage period for third-party donated oocytes, assuming that storage does not extend beyond reproductive age:

**Options:** 1 year / 2 years / 3 years / 4 years / 5 years / 10 years / Longer than 10 years

**4.** Please select one period that you consider appropriate as the maximum storage period for third-party donated sperm, assuming that storage does not extend beyond reproductive age:

**Options:** 1 year / 2 years / 3 years / 4 years / 5 years / 10 years / Longer than 10 years

**5.** Please select one period that you consider appropriate as the maximum storage period for embryos obtained from third-party donated sperm or oocytes, assuming that storage does not extend beyond reproductive age:

**Options:** 1 year / 2 years / 3 years / 4 years / 5 years / 10 years / Longer than 10 years

**6.** Please select one amount that you consider appropriate as monetary compensation for oocyte donors, excluding necessary costs related to medical care. If an applicable amount is not listed, please enter it under Other:

**Options:** No compensation / JPY 10,000 / JPY 50,000 / JPY 100,000 / JPY 300,000 / JPY 500,000 / Other: JPY ______

**7.** Please select one amount that you consider appropriate as monetary compensation for sperm donors, excluding necessary costs related to medical care. If an applicable amount is not listed, please enter it under Other:

**Options:** No compensation / JPY 1,000 / JPY 5,000 / JPY 10,000 / JPY 50,000 / JPY 100,000 / Other: JPY ______

**Section 6. Opinions Regarding Counseling and Telling**

When assisted reproductive technology using third-party donated gametes is performed, donors, recipients, and children born through donation treatment need opportunities to reflect carefully not only on circumstances and assumptions before treatment begins, but also on the possibility that treatment may have various long-term effects on emotions, relationships, and life. It is also necessary to provide information that once treatment begins and donation treatment is performed, pregnancy termination or relinquishment of childrearing may no longer be possible. Please answer what you consider appropriate regarding counseling and telling for donors and recipients.

**1.** International guidelines require support in the following areas as counseling for oocyte and sperm donors. Please answer whether each should also be implemented in Japan: donor psychological assessment support.

**Options:** Yes / No

**2.** Should donor decision-making support also be implemented in Japan?

**Options:** Yes / No

**3.** Should psychological support for donors during and after treatment also be implemented in Japan?

**Options:** Yes / No

**4.** International guidelines require support in the following areas as counseling for recipients of oocyte and sperm donation. Please answer whether each should also be implemented in Japan: In pre-treatment psychological assessment for recipients, caring for the grief of each spouse regarding infertility to date and discussing their thoughts about donation treatment and family formation.

**Options:** Yes / No

**5.** Should decision-making support to coordinate opinions among the couple and related parties also be implemented in Japan?

**Options:** Yes / No

**6.** Should mental health support during treatment, including helping recipients maintain appropriate expectations regarding treatment outcomes and providing psychological support when pregnancy is not achieved, also be implemented in Japan?

**Options:** Yes / No

**7.** Should support after treatment, during pregnancy, and during the postpartum and childrearing periods to promote bonding with the child and attachment from the child to the recipient, thereby supporting better childrearing, also be implemented in Japan?

**Options:** Yes / No

**8.** Should psychological support for forming parent-child and family relationships without a genetic connection through telling also be implemented in Japan?

**Options:** Yes / No

**9.** Counseling for recipient couples should proceed supportively while considering each spouse's understanding and feelings. Do you think counseling should be repeated not only through joint interviews with the couple, but also through separate opportunities for the husband and wife?

**Options:** Yes / No

**10.** Telling means more than simply disclosing who the biological parent is, whether there is a genetic connection, or how conception occurred. Rather, it means that the recipient couple repeatedly conveys love to the child and gives consideration so that the child can continue to live in this family with physical and emotional security and wellbeing. Do you think this kind of telling should also be practiced in Japan?

**Options:** Yes / No

**11.** In telling, if even non-identifying information about the donor, such as hobbies, special skills, or favorite school subjects at the time of donation, is not provided, it may be difficult for the child and recipient couple to form an image of, and sense of distance from, the donor without excessive expectations, excessive gratitude, or feelings of indebtedness toward the donor. If requested by the child, when and how much donor information should be disclosed to the recipient couple? Please select one option for each type of information: non-identifying donor information, such as hobbies, special skills, or favorite school subjects at the time of donation.

**Options:** At establishment of pregnancy / During pregnancy / After childbirth / When the child turns 3 years old / When the child turns ____ years old (please enter age) / When the child reaches adulthood / Do not provide any information regardless of age

**12.** Identifying donor information, such as name and address.

**Options:** At establishment of pregnancy / During pregnancy / After childbirth / When the child turns 3 years old / When the child turns ____ years old (please enter age) / When the child reaches adulthood / Do not provide any information regardless of age

**13.** When and how much donor information do you think recipient couples should tell the child born through donation? Please select one timing that you consider necessary overall, considering not only whether the child can understand words and meaning, but also the perspective of the recipient couple.

**Options:** At establishment of pregnancy / During pregnancy / After childbirth / When the child turns 3 years old / When the child turns ____ years old (please enter age) / When the child reaches adulthood / Do not provide any information regardless of age

Which institutions do you think should provide each of the following types of support?

- 1. Donor psychological assessment
- 2. Decision-making support for donor provision
- 3. Psychological support for donors during and after donation treatment
- 4. Care for each recipient spouse regarding grief over infertility to date
- 5. Support for each recipient spouse in organizing thoughts about donation treatment and family formation
- 6. Psychological assessment of each recipient spouse regarding donation treatment
- 7. Decision-making support regarding donation treatment for recipient spouses and related parties
- 8. Mental health support for recipients during treatment
- 9. Childrearing support for recipient couples after treatment, during pregnancy, postpartum, and during childrearing
- 10. Psychological support regarding telling to children born through recipient couples' donation treatment and the formation of parent-child and family relationships

**14.** Please select all support items from 1 to 10 that you think should be provided by The agency coordinating donation treatment. If an applicable item is not listed, please enter it under Other:

**Options:** 1 / 2 / 3 / 4 / 5 / 6 / 7 / 8 / 9 / 10 / None apply / Other: ____________

**15.** Please select all support items from 1 to 10 that you think should be provided by The medical institution that performs collection from donors. If an applicable item is not listed, please enter it under Other:

**Options:** 1 / 2 / 3 / 4 / 5 / 6 / 7 / 8 / 9 / 10 / None apply / Other: ____________

**16.** Please select all support items from 1 to 10 that you think should be provided by The medical institution that performs provision to recipients. If an applicable item is not listed, please enter it under Other:

**Options:** 1 / 2 / 3 / 4 / 5 / 6 / 7 / 8 / 9 / 10 / None apply / Other: ____________

**17.** If there are institutions other than those involved in infertility treatment or donation treatment that should be involved, please answer what kind of institutions they are. Please also select all support items from 1 to 10 that you think the institution should provide. If an applicable item is not listed, please enter it under Other:

**Options:** Institution type or name: ____________ / 1 / 2 / 3 / 4 / 5 / 6 / 7 / 8 / 9 / 10 / Other: ____________

This is the end of the questionnaire.

Reference

1. ESHRE Working Group on Reproductive Donation, Kirkman-Brown J, Calhaz-Jorge C, Dancet EAF, Lundin K, Martins M, Tilleman K, Thorn P, Vermeulen N, Frith L. Good practice recommendations for information provision for those involved in reproductive donation. Human Reproduction Open. 2022.
